# Bilateral Multifocal Serous Retinal Pigment Epithelial Detachments Coexisting With Generalized Rod-Cone Dysfunction Resembling Retinitis Pigmentosa

**DOI:** 10.7759/cureus.116855

**Published:** 2026-09-24

**Authors:** Hiromi Fukagai, Yuta Nariya, Koichiro Sugimoto, Shuichiro Aoki

**Affiliations:** 1 Ophthalmology, Kameda General Hospital, Kamogawa, JPN; 2 Ophthalmology, The University of Tokyo, Tokyo, JPN

**Keywords:** case report, electroretinography, fundus autofluorescence, generalized rod- cone dysfunction, multimodal imaging, retinal pigment epithelial detachment, serous pigment epithelial detachment

## Abstract

Bilateral multifocal serous retinal pigment epithelial detachment (PED) is uncommon, and its coexistence with generalized retinal dysfunction has rarely been reported. We report a 55-year-old man referred for bilateral fundus abnormalities who complained of a paracentral scotoma, photophobia, and nyctalopia. Best-corrected visual acuity was 20/16 in the right eye and 20/13 in the left eye. Fundus examination revealed numerous transparent, round serous PEDs distributed from the posterior pole to the equator in both eyes, with retinal pigment epithelium (RPE) atrophy adjacent to the vascular arcades. Optical coherence tomography showed multiple PEDs with hyporeflective lumens and outer retinal atrophy sparing the fovea. Fundus autofluorescence demonstrated hyperautofluorescent PED margins and hypoautofluorescent atrophic areas. Full-field electroretinogram (ERG) revealed severely reduced to non-recordable rod and combined rod-cone responses, with markedly reduced and delayed cone and 30-Hz flicker responses; Goldmann perimetry demonstrated bilateral ring scotomas. These findings indicated generalized rod-cone dysfunction resembling retinitis pigmentosa (RP). An extensive infectious and inflammatory workup was unremarkable, and no etiology was identified. The patient was managed conservatively and remained stable over two years of observation. This report documents a rare coexistence of bilateral multifocal serous PED and generalized rod-cone dysfunction resembling RP, characterized by multimodal imaging, full-field ERG, and Goldmann perimetry.

## Introduction

Retinal pigment epithelial detachment (PED) is the separation of the retinal pigment epithelium (RPE) from the inner collagenous layer of Bruch’s membrane. PEDs are categorized as serous, drusenoid, or vascularized based on their contents and multimodal imaging features [1,2]. In a serous PED, clear fluid accumulates beneath the RPE, producing a smooth, dome-shaped elevation. Serous PEDs most commonly develop secondary to other retinal conditions, particularly age-related macular degeneration (AMD) [1,2] and the pachychoroid disease spectrum, a group of disorders characterized by a thickened choroid that includes central serous chorioretinopathy (CSC) [3]. Bilateral multifocal serous PED, by contrast, is a rare and typically idiopathic condition in which numerous small serous PEDs are scattered throughout the posterior fundus of both eyes. It is considered distinct from AMD- or CSC-associated serous PED, and previously reported cases have generally followed a stable long-term course [4,5].

Retinitis pigmentosa (RP) is the most prevalent form of inherited retinal degeneration. Progressive dysfunction of rod photoreceptors, followed by cone photoreceptors, typically causes nyctalopia (night blindness), progressive visual field constriction, and a markedly reduced full-field electroretinogram (ERG) [6]. In previously reported cases of bilateral multifocal serous PED, by contrast, generalized retinal function assessed with full-field ERG has been normal in most tested patients [4,5]. Herein, we report a rare case of bilateral multifocal serous PED coexisting with generalized rod-cone dysfunction resembling RP. This functional pattern differs from the preserved retinal function in previously reported typical cases, and we discuss the differential diagnosis of this unusual combination of findings.

## Case presentation

A 55-year-old man was referred to our hospital after fundus abnormalities were detected during a routine health examination. Approximately one to two years before presentation, he became aware of a paracentral scotoma in his right eye. In April of the year of presentation, bilateral fundus abnormalities were noted at a local ophthalmic clinic, prompting referral to our hospital for further evaluation. He also reported long-standing nyctalopia and photophobia of insidious onset, present for many years, together with difficulty with dark adaptation. His medical history was notable for hypertension, and he had taken no medications associated with retinal toxicity, such as hydroxychloroquine. There was no family history of retinal disease.

At presentation, the best-corrected visual acuity was 20/16 in the right eye (-5.50 D sphere) and 20/13 in the left eye (-7.25 D sphere). Intraocular pressure was 15 mmHg in both eyes. Slit-lamp examination revealed mild cataracts in both eyes. No inflammatory findings, including anterior chamber cells or vitreous opacities, were observed.

Fundus examination revealed numerous transparent, round serous PEDs, measuring approximately 100-300 µm in diameter, distributed throughout the posterior pole and extending to the equatorial retina in both eyes. The lesions were particularly concentrated around the vascular arcades and the fovea. No exudative changes or retinal hemorrhages were observed. RPE atrophy was present adjacent to the vascular arcades, and localized retinal atrophy was noted in the inferior peripheral retina of the right eye (Figure 1). Fundus autofluorescence (FAF) demonstrated hyperautofluorescence along the margins of the PEDs, whereas areas of RPE atrophy showed hypoautofluorescence (Figure 2).

**Figure 1:**
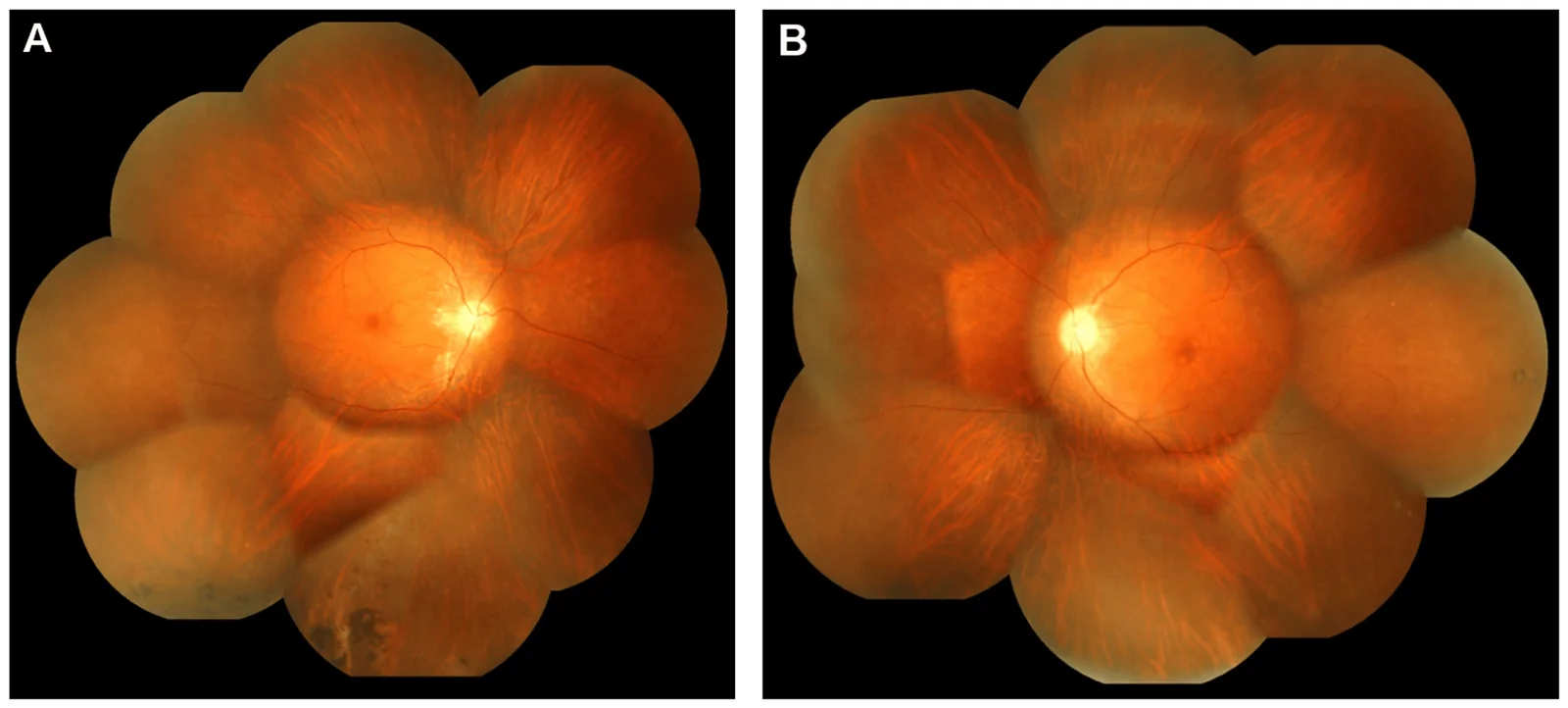
Ultra-widefield color fundus photographs of the right (A) and left (B) eyes. Numerous transparent, round serous retinal pigment epithelial detachments (~100-300 µm) are distributed from the posterior pole to the equator, concentrated around the vascular arcades and fovea. Retinal pigment epithelium atrophy is present adjacent to the arcades; localized inferior peripheral retinal atrophy is seen in the right eye.

**Figure 2:**
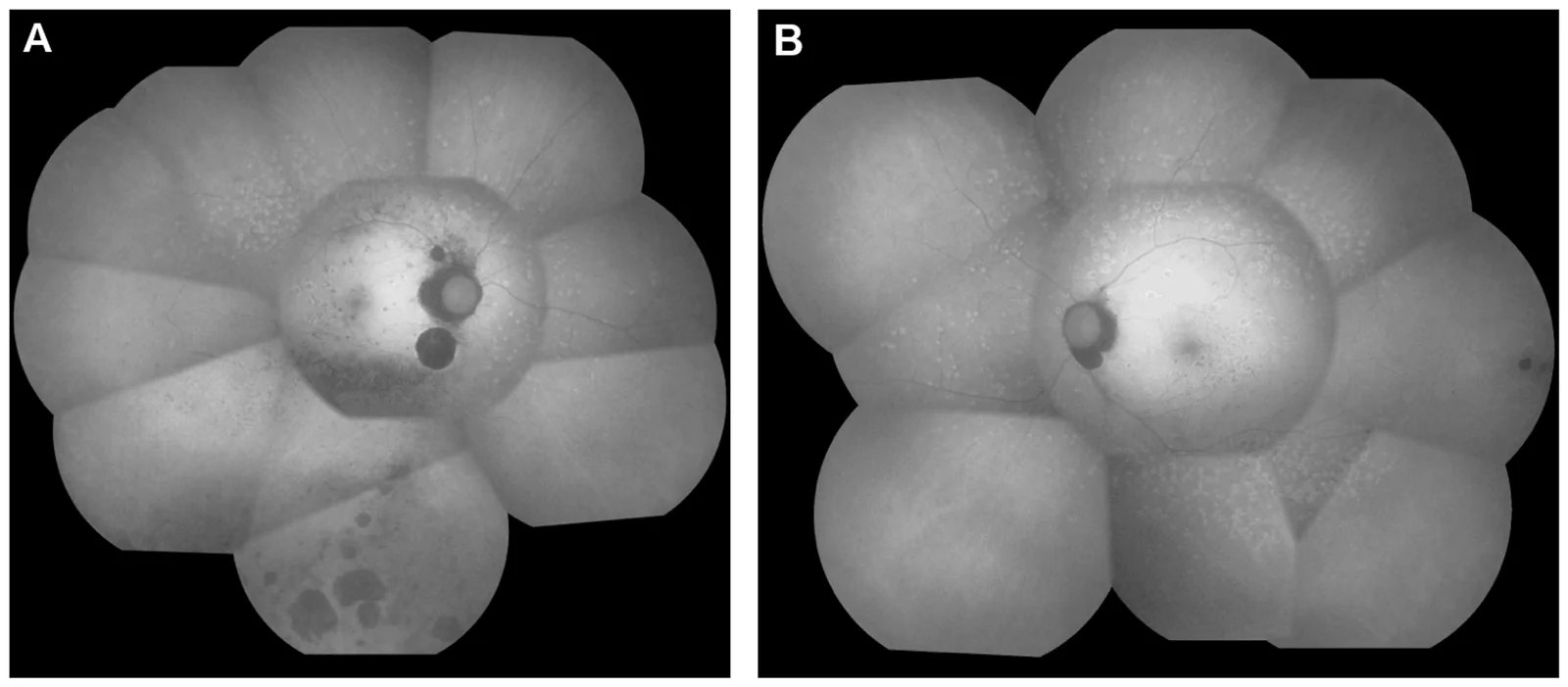
Fundus autofluorescence of the right (A) and left (B) eyes. The margins of the pigment epithelial detachments show hyperautofluorescence, whereas areas of retinal pigment epithelium atrophy show hypoautofluorescence.

Optical coherence tomography (OCT) revealed multiple PEDs with hyporeflective lumens and outer retinal atrophy across the parafoveal and perifoveal macula, whereas the subfoveal ellipsoid zone and outer retinal layers were preserved, consistent with the preserved central visual acuity (Figure 3). Fluorescein angiography (FA) demonstrated late pooling within the PEDs without fluorescein leakage or any findings suggestive of intraocular inflammation (Figure 4). Indocyanine green angiography (ICGA) demonstrated hypofluorescence corresponding to the PEDs, without choroidal hyperpermeability or choroidal neovascularization (Figure 5).

**Figure 3:**
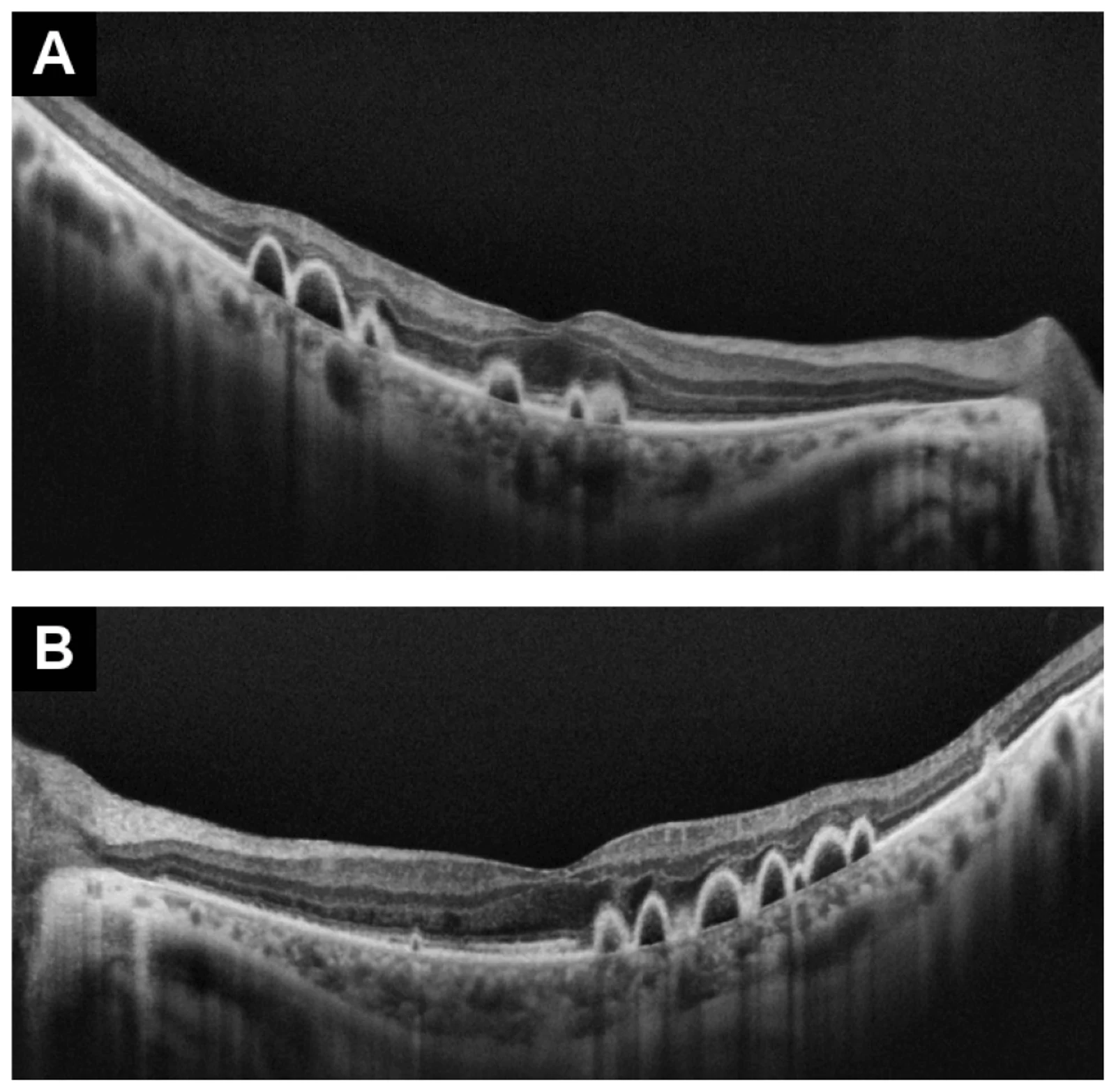
Optical coherence tomography of the right (A) and left (B) eyes. Multiple pigment epithelial detachments with hyporeflective lumens and outer retinal atrophy are seen across the parafoveal and perifoveal macula, with preservation of the subfoveal outer retina.

**Figure 4:**
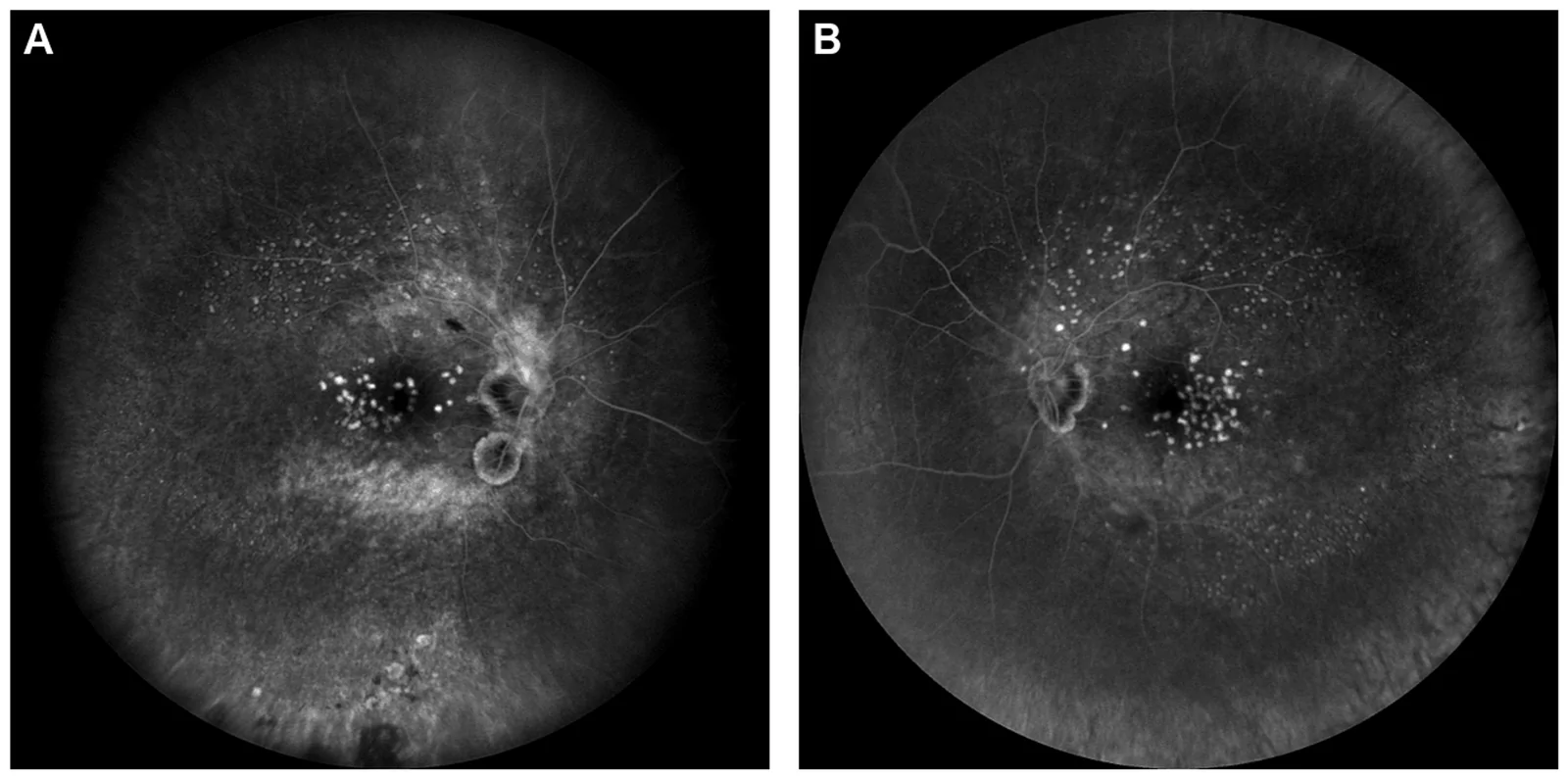
Ultra-widefield fluorescein angiography of the right (A) and left (B) eyes. Late pooling is seen within the pigment epithelial detachments, without fluorescein leakage or signs of intraocular inflammation.

**Figure 5:**
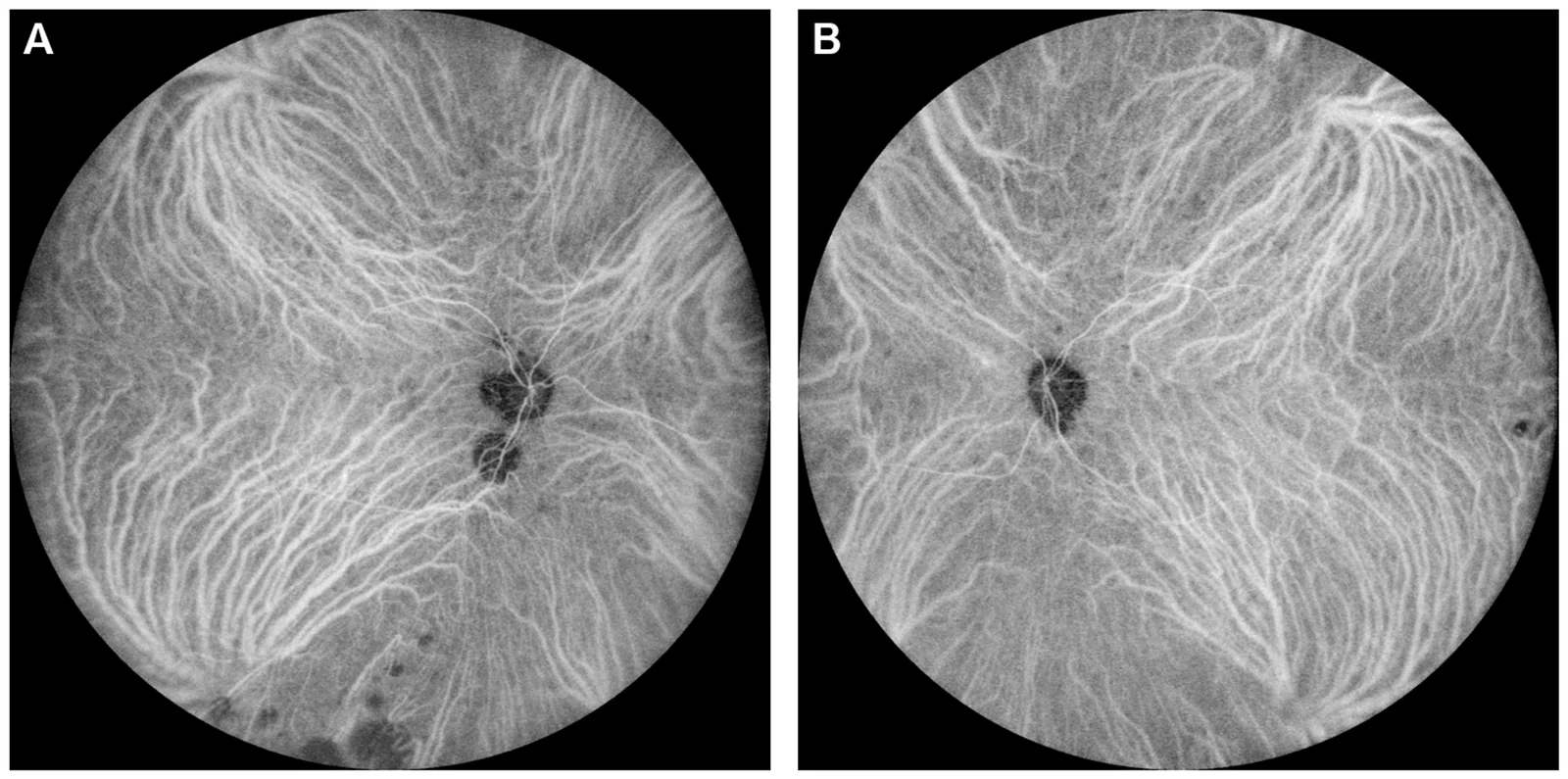
Ultra-widefield indocyanine green angiography of the right (A) and left (B) eyes. The pigment epithelial detachments appear hypofluorescent, without choroidal hyperpermeability or choroidal neovascularization.

Full-field ERG was recorded according to the International Society for Clinical Electrophysiology of Vision (ISCEV) standards [7]. The dark-adapted (DA) 0.01 response showed a severely reduced to non-recordable b-wave. The DA 3.0 response showed severely reduced a- and b-wave amplitudes with prolonged implicit times. The light-adapted (LA) 3.0 response and the LA 3.0 30-Hz flicker response showed markedly reduced amplitudes with prolonged implicit times. The rod-driven responses were more severely affected than the cone-driven responses, consistent with a rod-cone rather than a cone-rod pattern of generalized dysfunction (Figure 6). Goldmann perimetry demonstrated ring scotomas in both eyes (Figure 7).

**Figure 6:**
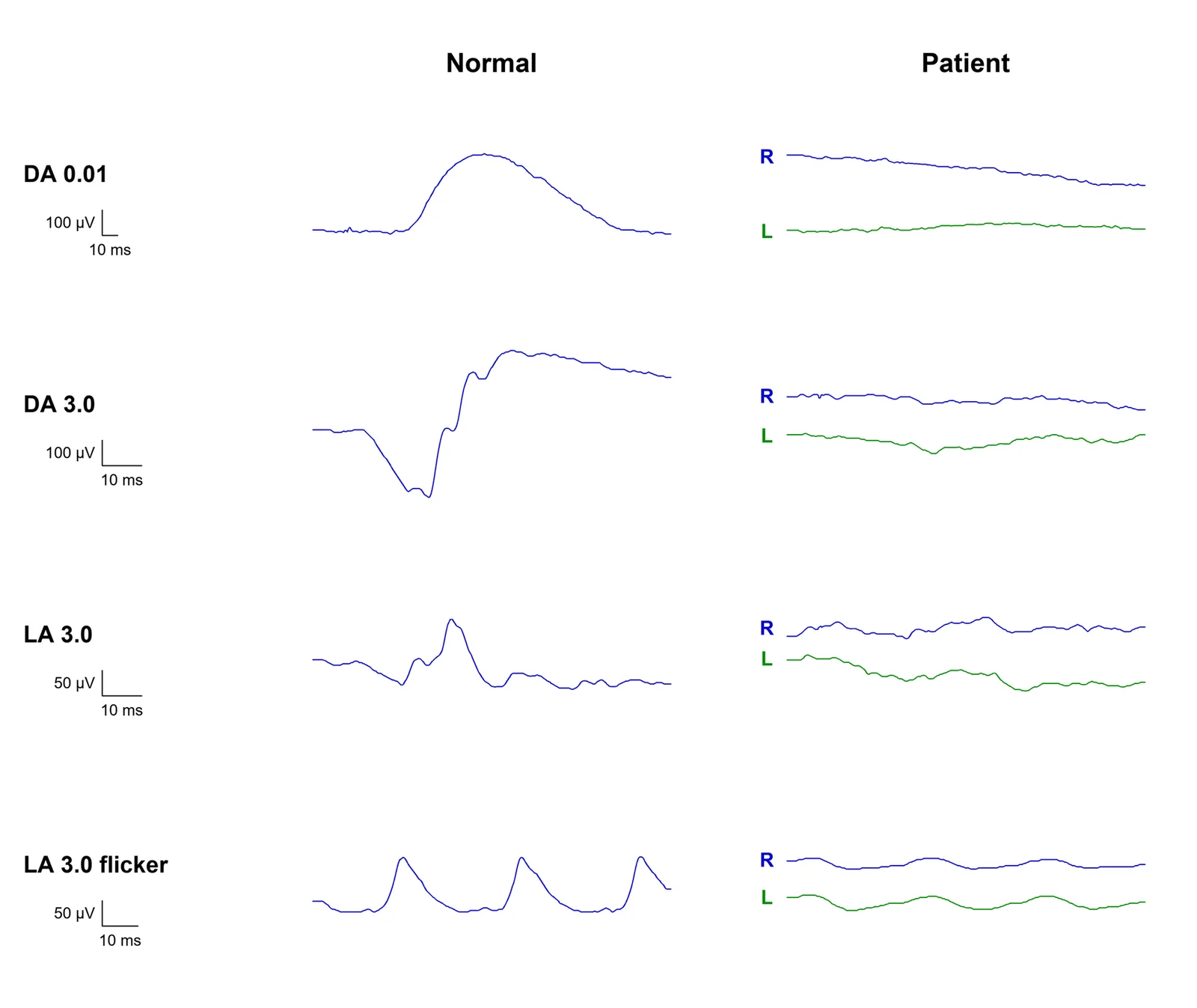
Full-field electroretinography (ERG) of the patient compared with a normal control. Recorded according to International Society for Clinical Electrophysiology of Vision (ISCEV) standards. From top to bottom: dark-adapted (DA) 0.01 (rod), DA 3.0 (combined rod-cone standard-flash), light-adapted (LA) 3.0 (cone), and LA 3.0 30-Hz flicker responses. Left column, a representative normal control (one eye); right column, the patient, with the right eye (R, blue) above the left eye (L, green). The DA 0.01 and DA 3.0 responses are severely reduced to non-recordable, and the LA 3.0 and LA 3.0 30-Hz flicker responses are markedly reduced with prolonged implicit times, indicating generalized rod-cone dysfunction.

**Figure 7:**
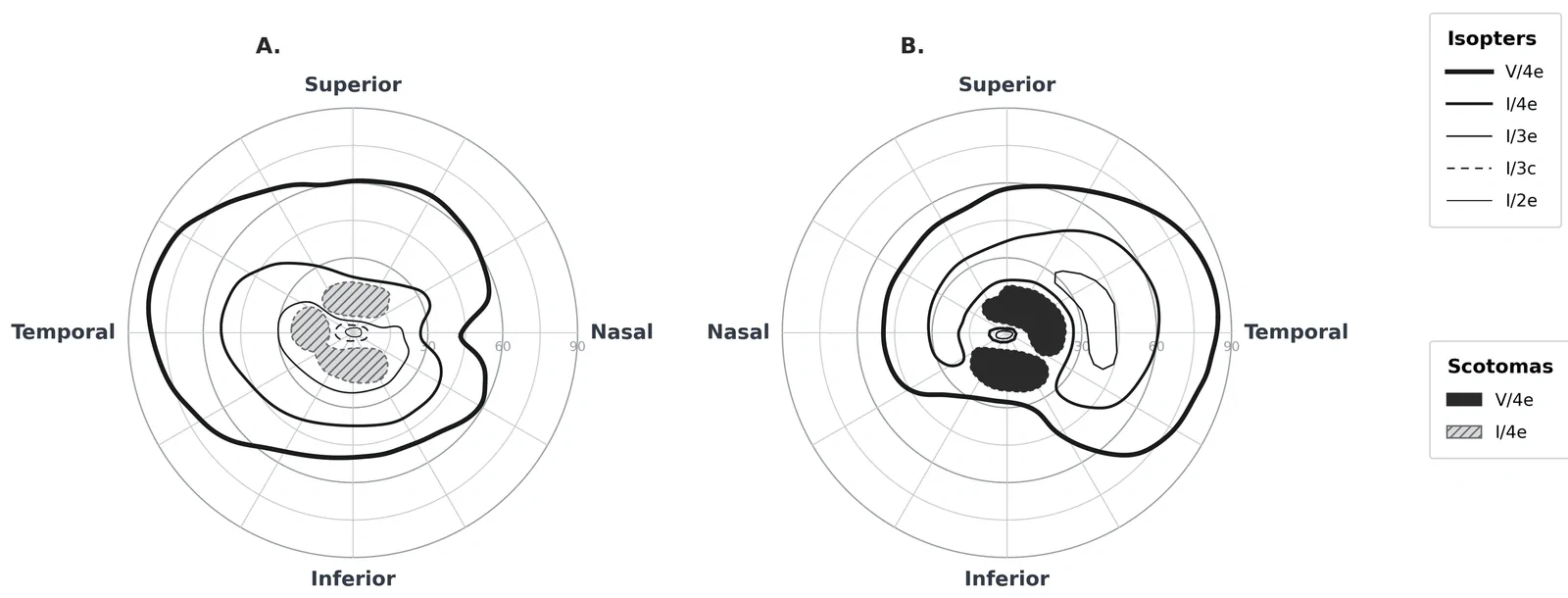
Goldmann kinetic perimetry of the left eye (OS) (A) and right eye (OD) (B). Bilateral ring scotomas are demonstrated.

Laboratory evaluation revealed normal liver and renal function, normal inflammatory markers, and a negative antinuclear antibody test. Serologic testing for herpes simplex virus, varicella-zoster virus, cytomegalovirus, human T-cell leukemia virus type 1, syphilis, and toxoplasmosis was also negative.

The patient was diagnosed with generalized rod-cone dysfunction resembling RP coexisting with bilateral multifocal serous PEDs, although the underlying etiology could not be determined. Because the PEDs were not associated with hemorrhage or exudative changes, the patient was managed conservatively with regular follow-up. Over two years of observation at our institution, best-corrected visual acuity, subjective symptoms, and fundus findings remained unchanged.

## Discussion

Bilateral multifocal serous PEDs constitute an uncommon clinical entity characterized by numerous small PEDs distributed throughout the fundus. Although previous reports suggest that the condition usually presents bilaterally from young adulthood to middle age and often follows a relatively stable course over many years, only a limited number of cases have been reported, and its pathogenesis and long-term prognosis remain poorly understood [4,5,8-12]. Choroidal neovascularization has occasionally been reported as a complication of this entity [4,11]. In the reported cases, full-field ERG and electro-oculography were normal in most tested patients [4,5], and in one case with 16-year follow-up, the full-field ERG remained normal, with only localized multifocal ERG attenuation, even as the PEDs evolved morphologically [13]. The markedly abnormal full-field ERG, combined with the bilateral ring scotomas and outer retinal atrophy on OCT, is inconsistent with the typical idiopathic entity and instead points to a coexisting generalized retinal degeneration, rather than the PEDs themselves.

Several alternative diagnoses were considered. CSC should be considered in the differential diagnosis of serous PED; however, the multifocal, bilateral, non-exudative PEDs with hyperautofluorescent margins seen here differ from typical CSC, and neither choroidal thickening on OCT nor choroidal hyperpermeability on ICGA was observed [14]. Large colloid drusen and cuticular drusen may present with a similar multifocal fundus appearance. Characteristic imaging features of these drusen include sub-RPE deposits on OCT and, on FAF, a target-like pattern of central hypoautofluorescence with a hyperautofluorescent rim in cuticular drusen [15-18]. Notably, the nosological boundary between bilateral multifocal serous PED and cuticular or large colloid drusen remains debated. Gass et al. originally described multifocal serous PED as a distinct entity [4], whereas a recent long-term report proposed that the two lie along a single continuum: in that case, hyporeflective serous PEDs evolved into hyperreflective, colloid-like sub-RPE material over 16 years, prompting reclassification of the phenotype as a drusen subtype, although this rests on a single longitudinal case [13]. Because our patient’s PEDs remained serous and hyporeflective without solid sub-RPE deposits, we did not classify them as large colloid drusen. Pattern dystrophy and adult-onset vitelliform lesions can also produce multifocal sub-RPE deposits, but the absence of vitelliform material and the autofluorescence pattern seen here argued against these entities. Moreover, full-field ERG is typically normal in pattern dystrophy, whereas our patient showed markedly abnormal responses, further arguing against this diagnosis [19].

Other mimics were also considered. Acute zonal occult outer retinopathy can likewise cause scotomas contiguous with the blind spot, outer-retinal dysfunction, and an abnormal ERG with preserved acuity, but its characteristically zonal outer-retinal involvement, which is frequently asymmetric, contrasts with the symmetric, generalized rod-cone dysfunction observed here [20,21]. Autoimmune and paraneoplastic retinopathies can produce a markedly reduced ERG with relatively preserved central acuity, a pattern that overlaps with the present case; antiretinal antibody testing and malignancy screening were not performed, and this possibility could not be formally excluded.

In addition to the multifocal serous PEDs, our patient demonstrated features characteristic of generalized rod-cone dysfunction resembling RP, including a markedly abnormal full-field ERG, outer retinal atrophy sparing the fovea, and bilateral ring scotomas [6,22,23]. Despite this generalized dysfunction, central visual acuity was preserved, in keeping with the relative foveal sparing seen on OCT. Abnormal multifocal electroretinographic responses have previously been reported in association with multifocal PEDs, suggesting that photoreceptor and RPE dysfunction may co-occur in this setting [11]. A serous PED reflects the accumulation of fluid beneath the RPE and is generally attributed to choroidal hyperpermeability or altered hydraulic resistance at the level of Bruch's membrane rather than to primary photoreceptor or RPE dysfunction; indeed, serous PED is not a typical feature of other retinal degenerations in which RPE function is impaired. In the present case, choroidal hyperpermeability was not evident on indocyanine green angiography, and we therefore do not propose a direct causal mechanism linking the retinal degeneration to the serous PEDs. Whether the multifocal serous PEDs and the generalized rod-cone dysfunction in our patient are causally related or represent coincidental comorbidity cannot be determined from a single case.

This report has limitations. Genetic testing and antiretinal antibody testing were not performed, which limits definitive etiologic classification of the generalized rod-cone dysfunction and precludes formal exclusion of autoimmune retinopathy; the stable two-year course, however, would be atypical for paraneoplastic retinopathy. Because the natural history of this combined presentation is unknown, long-term follow-up is warranted, with particular attention to progression of the retinal degeneration, possible evolution of the serous PEDs toward a colloid-drusen morphology, and the development of choroidal neovascularization.

## Conclusions

We report a rare case of bilateral multifocal serous PEDs coexisting with generalized rod-cone dysfunction resembling RP, of undetermined etiology. The diagnosis was based on the integration of multimodal imaging and full-field ERG findings and supported the presence of generalized rod-cone dysfunction resembling RP. In this patient, a markedly abnormal full-field ERG and bilateral ring scotomas distinguished coexisting generalized rod-cone dysfunction from typical idiopathic multifocal serous PEDs, in which generalized retinal function is preserved. The undetermined etiology and unknown natural history of this combination call for continued long-term observation.
